# Infectious complications during monoclonal antibodies treatments and cell therapies in Acute Lymphoblastic Leukemia

**DOI:** 10.1007/s10238-023-01000-9

**Published:** 2023-01-30

**Authors:** Martina Quattrone, Alessia Di Pilla, Livio Pagano, Luana Fianchi

**Affiliations:** 1 Dipartimento di Diagnostica per Immagini, Radioterapia Oncologica ed Ematologia, UOC Ematologia Geriatrica ed Emopatie rare, Università Cattolica del Sacro Cuore, Rome, Italy; 2 Fondazione Policlinico Universitario Agostino Gemelli – IRCCS, Rome, Italy

**Keywords:** Monoclonal antibodies, Acute Lymphoblastic Leukemia

## Abstract

Infections represent one of the most frequent complications during the treatment of patients with Acute Lymphoblastic Leukemia (ALL): of these, almost half develop an infectious event in the majority of cases in induction. The new monoclonal and bispecific antibodies and CAR-T, besides offering new perspectives in the overall survival and disease-free survival of patients, may also transform the epidemiology of infections in ALL by improving the toxicity of treatments. In this review, we examined studies published in the literature over the past 12 years and described the infectious complications of therapy with Blinatumomab, Inotuzumab, Rituximab and CAR-T in adult and pediatric patients with ALL. Infections are less frequent than in traditional chemotherapy treatment with vincristine, corticosteroids and anthracyclines, which has been the backbone of therapy for patients with ALL for years. On the other hand, the infection scenario in the CAR-T setting is quite peculiar: In these patients, infections are more frequent in the first month after infusion and are predominantly bacterial. As the time moves away from day zero, viral infections become more frequent, occurring mainly in patients who have had prolonged cytopenia and major cytokine release syndrome.

## Introduction

Acute Lymphoblastic Leukemia (ALL) is a malignant transformation and proliferation of lymphoid progenitor cells in the bone marrow, blood and extramedullary sites. Despite advances in management, the backbone of ALL therapy remains multi-agent chemotherapy with vincristine, corticosteroids and an anthracycline with allogeneic stem cell transplantation for eligible candidates. Infections, unfortunately, are one of the most relevant adverse events of traditional chemotherapy, and they have a strong impact on patients’ overall survival [1–3]. On the other hand, monoclonal antibody-based therapies combine their high efficacy with a lower burden of infectious adverse events, representing an important resource in ALL treatment [1–3].

Among patients with ALL, 45% experience infectious complications during the course of the disease [4]. Most of the infections in ALL patients are bacterial (40%), mostly gram-negative, and they are more frequent during induction therapy: 53% of patients experience and infectious complication during induction therapy and 44.3% at relapse. Refractory patients have the highest incidence of infection rate (85.7%), whereas patients in complete remission the lowest (35.7%) [4].

Bacterial infections occur in 28% of cases during induction, 19% during either consolidation or maintenance and in 23% of refractory patients. Although bacteria are the most frequently involved microorganism, bacterial infections seem to have little impact on overall and infection mortality, as compared to invasive fungal infection and mixed infections [4].

Fungal infections are responsible of 9% of infections in ALL patients, and mold is more common than yeast [4]. Invasive aspergillosis and candidemia are more common in refractory patients than in those who achieve remission (28.5% vs. 1.3%), suggesting a crucial role of the immune system reconstitution [4].

Viral infections are not as common in ALL (2%), but their incidence could be underestimated, considering they are often subclinical. They are more frequent in relapsed (6%) and refractory patients (14%) [4].

Mixed infections caused primarily by bacteria and fungi accounts for 6% of infections in ALL and they are responsible for the highest mortality rates. The presence of mixed infections likely reflects mucosal damage observed in leukemia patients undergoing chemotherapy, and they are more frequent in relapsed/refractory patients (14%) [4].

Despite the improved diagnostic procedures, fever of unknown origin (FUO) represents a considerable part of infectious complications in ALL patients. FUO is defined as a temperature greater than 38.3 °C on several occasions, more than 3 weeks’ duration of illness, and failure to reach a diagnosis despite one week of inpatient investigation. There is still controversy if FUO are undetected infectious episodes or phenomena related to the hematologic malignancy or its treatment. In any case, mortality rates registered during FUO are low (3.6%). Probably prompt empiric prescription of anti-infective drugs at fever onset might play a role in the reduction of mortality rates for infections misdiagnosed as FUO and that are sustained instead by microorganisms. In the literature, the incidence of FUO in ALL is 53% in the induction phase, 36% during consolidation therapy and 44% in relapsed or refractory patients [5].

In children, outcome in Acute Lymphoblastic Leukemia has definitely shown an improvement, with recent trials demonstrating excellent survival in standard-risk patients. However, treatment-related toxicity remains unacceptably high, with infections, in particular, being the most important cause of treatment-related mortality [6].

Infectious events, indeed, account for approximately 30% of deaths in the pediatric population with ALL. As a result, it is critical to address infections to further improve disease prognosis, especially in the low-risk patients [7].

Among the patients most prone to the incidence of infectious events are primarily those with Down syndrome and, to a lesser extent, female patients and those on higher intensity chemotherapy regimens, while the phase of treatment course when infection is most likely to occur is induction (77% vs. 56% during consolidation), particularly during neutropenia [8]. In 45% of cases, patients develop a FUO, in 68% of cases bacterial infections were reported, most frequently due to Gram negatives as *Pseudomonas spp*, *E. coli* and *Enterococcus.* Fungal infections are documented in 20% of cases in pediatric patients, and the most frequent identified fungal agent is *Aspergillus*; among viruses, which account for 12% of infections, the most important causes are represented by *Respiratory syncytial virus* and *Varicella Zoster *[8]*.*

In this review, we offer perspectives on the infectious complications of monoclonal antibodies treatments and cell therapy in Acute Lymphoblastic Leukemia. Specifically, we provide an overview of the infectious risk related to the use of Blinatumomab, Inotuzumab ozogamicin and Rituximab, either monotherapy or in combination, and CAR-T cells therapy in patients with ALL. Our goal is to provide a solid background of scientific data to stratify by risk patients treated with monoclonal antibodies and cellular therapy, to improve infection prophylaxis and treatment.

## Material and methods

We performed a review of the literature published in the last 12 years regarding infectious complications in patients with Acute Lymphoblastic Leukemia at any stage of disease, treated with new monoclonal antibodies or cell therapies.

Specifically, we analyzed the infectious complications associated with the use of Blinatumomab, Inotuzumab ozogamicin and Rituximab used in monotherapy or in combination with other treatments and infectious events that occurred after infusion of CAR-T cells in patients with Acute Lymphoblastic Leukemia. Moreover, we stratified infections according to the microorganisms responsible and the phase of the disease.

Our data were reported in tables summarizing the most frequent infectious complications of the treatments analyzed and subdividing them according to pathogenic noxa (bacterial, viral, fungal) and clinical presentation (febrile neutropenia, pneumonia, sepsis).

## Blinatumomab

Blinatumomab is a CD19 BiTE (bispecific T-cell engager) immuno-oncology therapy that activates endogenous cytotoxic T cells to kill target B cells. Blinatumomab is indicated for the treatment of adults and children with relapsed/refractory (R/R) B-cell precursor (BCP) ALL and for patients with minimal residual disease (MRD) ALL, defined as at least 10^−3^ (0.1%) leukemic cells detected by quantitative polymerase chain reaction (PCR)**.**

The randomized phase 3 TOWER trial compared Blinatumomab with conventional chemotherapy as salvage treatment in relapsed/refractory patients with Philadelphia-negative ALL [9]. In this study, Blinatumomab was shown to be associated with longer overall survival and higher rates of complete remission compared to standard chemotherapy. Patients treated with Blinatumomab also achieved MRD negativity significantly more frequently than conventional chemotherapy (76 vs. 48%) [9].

In terms of infectious complications, in the TOWER study 52.8% infections were reported during the induction phase, 75% during consolidation and 58.3% during maintenance therapy. Interestingly, only 16.7% of the infectious events in induction phase were of grade three or higher, whereas during consolidation and maintenance this percentage reached 22.2% [9].

In Table 1, we reported the infectious complications described in the main studies that analyzed the efficacy and safety of Blinatumomab in pediatric and adult patients with Acute Lymphoblastic Leukemia.

**Table 1:** Use of Blinatumomab and infectious events

References	Study	Monotherapy/combination	Patients (n°)	Phase of disease	Febrile neutropenia/FUO	Pneumonia	Sepsis	Bacterial infection	Fungine	Viral	Others
Topp et al. [15]	Phase 2	Monotherapy	21, adults	MRD positive		1 (5%)	1 (5%)	Device-related: 2 (10%)			
Topp et al. [10]	Phase 2	Monotherapy	189, adults	Relapsed/refractory	53 (28%)	18 (10%)	13 (7%)		4 (2%)		
Kantarjian et al. [9]	Phase 3	Monotherapy	267, adults	Relapsed/refractory	64 (24%)	16 (6%)	14 (5%)	Device related 16 (6%)	Aspergillosis 4 (1.5%) Fungal sepsis 2 (1%) Lower respiratory tract infection 1 (0.4%)	Oral *Herpes simplex* 15 (6%) Encephalitis enteroviral 1 (0.4%) *Influenza* 1 (0.4%)	Upper respiratory tract infection 19 (7%)
Gökbuget et al. [16]	Phase 2	Monotherapy	116, adults	MRD positive	18 (16%)*			Device-related 8 (7%) Staphylococcal 4 (3%) UTI 6 (5%)			
Stein et al. [12]	Phase 2	Monotherapy	64, adults	After allo-HSCT	13 (20%)	6 (9%)					
Foà et al. [13]	Phase 2	With Dasatinib	63, adults	After induction with Dasatinib						*CMV* reactivation: 6 (10%)	
Rambaldi et al. [17]	Phase 3	Monotherapy	267**, adults	Relapsed/refractory		1 (3%)	1 (3%)	Device-related 2 (6%) *C.Difficile* 1 (3%) Mastoiditis 1 (3%) UTI 1 (3%)		PML 1 (3%) *HZV* 1 (3%)	
Martinelli et al. [14]	Phase 2	Monotherapy	45, adults	Relapsed/refractory	5 (11%)						
Brown et al. [11]	Phase 3	Monotherapy	102, pediatric	Relapsed/refractory	6 (5.9%)		2 (2%)				

*HZV* Herpes Zoster Virus, *MRD* minimal residual disease, *PML* progressive multifocal leukoencephalopathy and *UTI* upper respiratory tract infection

^*^neutropenia with or without fever

^**^Blinatumomab randomization = 271 patients; consolidation = 86; maintenance = 36

The incidence of infections is quite variable in the different studies, probably also depending on the type of patients considered (age, comorbidity), the stage of the disease (morphological progression, positive MRD, complete remission during consolidation or maintenance phase) and the treatment schedule (induction, consolidation or maintenance with Blinatumomab and possible association with other drugs).

Of the studies reviewed, the phase 2 study by Topp et al. [10] reported the largest number of infectious complications: 28% of patients developed febrile neutropenia, 10% pneumonia and 7% sepsis. In addition, 2% of patients developed fungal infections, in particular *Fusarium, Aspergillus* and *Candida* infections [10].

This study was conducted in adult patients (> 18 years old), who were either primary refractory after induction or who had relapsed within 12 months of first remission, relapsed within 12 months of receiving allogeneic hematopoietic stem cell transplantation (allo-HSCT), or not responded to or relapsed after first salvage therapy or beyond [10]. Of these, 39% had received at least two lines of salvage therapy and 34% had previously received an allo-HSCT [10].

The study in which infectious complications was less frequent was the phase 3 trial by Brown et al. [11], conducted in a population of pediatric and young adult patients with ALL in first relapse after induction treatment.

Brown et al. [11] compared Blinatumomab to conventional chemotherapy: 27% of infectious events occurred in patients treated with Blinatumomab, compared to 65% in patients treated with chemotherapy. In particular, 5.9% of patients treated with Blinatumomab experienced febrile neutropenia (vs. 57.7%) and 2% developed sepsis (26.8%).

Kantarjian et al. [9] also compared the efficacy and toxicities of Blinatumomab with those of conventional chemotherapy: 24% of patients treated with Blinatumomab developed febrile neutropenia versus 39% of patients receiving chemotherapy, 7% developed upper respiratory tract infections (vs. 1% in the chemotherapy group), 6% developed catheter-related infections (vs. 5.5%), 6% developed pneumonia (vs. 14.7%) and 5.2% developed sepsis (vs. 7.3%).

Stein et al. [12] with their phase 2 study published in 2019, studied patients with ALL who received Blinatumomab as salvage therapy for relapsed after allo-HSCT. In these patients, 20% developed febrile neutropenia and 9% developed pneumonia. The authors did not describe a particular increase in viral or fungal infections in their cohort [12].

A peculiar therapeutic scenario in which to place treatment with Blinatumomab is that of Philadelphia-positive ALL [13, 14]. In one study, in particular, the most frequent infectious complication in patients who had undertaken combination treatment with Blinatumomab and Dasatinib was *Cytomegalovirus* reactivation, which affected 10% of patients [13]. In contrast, in another study, in which Blinatumomab was administered as monotherapy, no increased incidence of viral infections was observed, although 11% of patients developed febrile neutropenia [14].

With regard to the most common etiological agents involved in the infections of patients treated with Blinatumomab, it is clear from studies published in recent years that the most frequent infectious complications are bacterial: up to 10% of patients developed bacterial pneumonia, 2–7% of patients developed sepsis and 6–10% of patients developed device-related infection [9–17]. Fungal infections, on the other hand, are less frequent, although they are often characterized by significant morbidity and mortality [9, 10]. Aspergillosis (1.5%), fungal sepsis (1%) and fungal pneumonia (0.4%) were described [9, 10].

With regard to viral infections, the etiological agents most frequently responsible for infectious scenarios are those of the *Herpes* family: *Herpes simplex virus 1*6%, *Herpes Zoster Virus* 1% [9, 13, 17]. On the other hand, *Enterovirus* (0.4%) and *Influenza* (0.4%) occurred less frequently; a very dangerous complication is represented by progressive multifocal leukoencephalopathy (PML) which account for 3% of cases and it is caused by the combination of *JCV* infection and deep immunosuppression [9, 13, 17].

## Inotuzumab ozogamicin

Inotuzumab ozogamicin is a monoclonal antibody against CD22 that is bound to calicheamicin, a toxic natural product of *Micromonospora echinospora*. Calicheamicin binds to the minor DNA groove and causes breaks in double-stranded DNA in a sequence-specific and thiol-dependent manner and leads to cell apoptosis.

In Table 2, we have listed the most frequently described infectious complications in the literature in patients treated with Inotuzumab [18–23].

**Table 2:** Use of Inotuzumab ozogamicin and infectious events

References	Study	Monotherapy/combination	Patients (n°)	Phase of disease	Infections	Febrile neutropenia/FUO	Pneumonia	Sepsis	Bacterial infection	Fungine	Viral
Kantarjian et al. [18]	Phase 2	Monotherapy	49, adults and children	Relapsed/refractory		8 (16%)	5 (10%)	8 (16%)		2 (4%)	5 (10%)
Kantarjian et al. [19]	Phase 3	Monotherapy verses standard PCT	139, adults	Relapsed/refractory	29 (21%)	16 (12%)	5 (4%)	6 (4%)	*C*. *Difficile* 2 (1%)	0	
Jabbour et al. [20]	Phase 2	Combined*	48, adults	Relapsed/refractory	32 (67%)			1 (2%)			
Kantarjian et al. [21]	Phase 2	Combined with low intensity PCT**	52, adults	Onset	48 (92%)						
DeAngelo et al. [22]	Phase 3	Monotherapy verses standard PCT	162, adults	Relapsed/refractory		9 (17%) 17 (22%) 16 (53%)	2 (4%) 2 (3%) 1 (3%)	1 (2%) 1 (1%) 0			
Brivio et al. [23]	Phase 1	Monotherapy	25, pediatric	Relapsed/refractory (pediatric)		8 (32%)					Rhinitis: 3 (12%)

*Combined with mini-hyper-CVD with or without Blinatumomab

**Mini-hyper-CVD

The incidence of infections in patients undergoing treatment with this monoclonal antibody varies depending on the treatment regimen (Inotuzumab as monotherapy vs. in combination), the type of patients enrolled and the stage of the disease (92% infections at onset vs. 67% in relapsed/refractory patients) and the burden of disease [18–23].

According to data, 3–10% of patients had a bacterial lung infection and up to 16% developed sepsis. Viral infections are fairly frequent (around 10% of cases), while fungal infections are uncommon (4%) [18–23].

The phase three INO-VATE study, which led to the market approval of this drug, compared the efficacy and safety of Inotuzumab ozogamicin with that of conventional chemotherapy in a cohort of adult patients with relapsed or refractory ALL [19]. This comparison showed a lower overall incidence of infections in patients receiving Inotuzumab than in those receiving chemotherapy (21% vs. 31%), and a reduced incidence of febrile neutropenia (12% vs. 18%), sepsis (4% vs. 8%) and fungal infections (0% vs. 2%) [19]. Pneumonia, on the other hand, was described more frequently in patients treated with Inotuzumab (4% vs. 1%); no particular incidence of viral infections was described [19].

In a more recent study, the efficacy and safety of Inotuzumab monotherapy in pediatric patients were described [23]. Surprisingly, a high incidence of febrile neutropenia (32%) but no incidence of bacterial infections or sepsis was reported. Collaterally, 12% of patients developed an upper airway infection [23].

Many studies have described the use of Inotuzumab with other chemotherapeutic drugs [20, 21]: In these studies, a high number of febrile neutropenia was reported, between 67 and 92%. Probably the toxicity of the drugs used in the combination scheme merges with that of Inotuzumab, making infectious complications more frequent, although rigorous comparison studies would be needed for a more conclusive confrontation [20, 21].

One of the factors that seems to have an impact on the efficacy and safety of Inotuzumab is certainly the disease burden. In a recent study, patient was divided into 3 classes according to disease burden and infections had a higher incidence in patients with the highest burden [22]. Specifically, 53% of patients with high burden developed febrile neutropenia, compared to 21.5% and 17% of patients with lower burden [22]. In the same study, patients receiving standard chemotherapy treatment were compared to those treated with Inotuzumab as monotherapy [22]. In patients receiving standard chemotherapy treatment, the incidence of febrile neutropenia was 55.8%, 54.9% and 46.4% in classes of patients with an increasing burden of disease [22]. It is thus noted that on average the incidence of febrile neutropenia is higher in patients treated with chemotherapy than in those treated with Inotuzumab, and that in the latter the incidence of febrile neutropenia appears to be more influenced by disease burden than in patients who received chemotherapy [22].

## Rituximab

Rituximab is a genetically engineered chimeric mouse/human monoclonal antibody representing a glycosylated immunoglobulin with human IgG1 constant regions and murine light chain and heavy chain variable region sequences, targeting cells CD20 positive.

Table 3 shows the most recent data in the literature on the epidemiology of infections in patients with ALL treated with Rituximab [24–27].

**Table 3:** Use of Rituximab and infectious events

References	Study	Monotherapy/combination	Patients (n°)	Phase of disease	Infections	Febrile neutropenia/FUO	Pneumonia	Sepsis	Bacterial infection	Fungine	Viral
Intermesoli et al. [24]	Phase 2	Combined with PCT	105, adults	Onset	51 (49%)		14 (13%)	27 (26%)			
Ribera et al. [25]	Phase 2	Combined with PCT	118, adults	Onset	30%						
Hoelzer et al. [26]	Phase 4	Combined with PCT	265, adults	Onset	22%						
Maury et al. [27]	Phase 3	Combined with PCT	105, adults	Onset	71 (68%)			45 (43%)		Candidemia 1 (1%) Aspergillosis 1	

The incidence of infection in patients receiving Rituximab is highly variable and depends on the stage of treatment, the other drugs used in combination, and the age and biological characteristics of the patients, with rates varying from 22 to 70% of infectious events in the different studies [24–27].

Regarding infectious agents, in most cases infections in patients treated with Rituximab are bacterial, particularly lower airway infections (17%) and sepsis (31–43%). In contrast, fungal infections, such as aspergillosis (1%) and candidemia (1–2%), are rare [24–27].

Rituximab use has been studied mainly in combination with other drugs, which certainly influence its toxicity profile and thus also its infectious risk.

In a recent study, Rituximab was administered to more than 100 adult patients with ALL, in combination with high-dose methotrexate, fractionated ifosfamide/cyclophosphamide, other drugs in rotation and intrathecal chemoprophylaxis [24]. In this study, 49% of patients developed infections, with a significantly higher incidence in older patients [24]. The most frequently reported infections in the study were bacterial pneumonia (13%) and sepsis (26%) [24]. Considerably inferior, however, was the prevalence of infections in other studies of adult patients treated with intensive chemotherapy combined with Rituximab: In that case only 22% of patients developed an infection [26].

If we consider *HIV*-positive patients, the incidence of infections is approximately 30% when Rituximab is administered with intensive chemotherapy [25].

Since Rituximab is mostly used in combination with other drugs, it is difficult to estimate the infectious risks that depend strictly on this drug and instead are caused by intensive chemotherapy alone. In their study, Maury et al. [27] divided patients into two groups based on whether or not Rituximab was added to the treatment schedule and analyzed efficacy and safety. In the group of patients treated with chemotherapy, 53% of patients developed an infectious event, while in patients who received chemotherapy and Rituximab, the infection rate was 68% [27]. Notably, 43% of patients who were treated with chemotherapy and Rituximab developed sepsis, while only 32% of patients who did not perform Rituximab therapy experienced this complication [27]. Regarding fungal infections, there were no substantial differences between the two groups: 1% of patients developed Candidemia among both Rituximab and intensive chemotherapy-only patients, while 1% of Rituximab-treated patients developed invasive Aspergillosis (vs. 0% in the control group) [27].

## Chimeric antigen receptor (CAR) T cell immunotherapy

CAR-T cells are engineered T lymphocytes, obtained by leukapheresis and genetically modified by introducing viral carriers into competent T lymphocytes. This process leads to the expression of a chimeric transmembrane receptor (CAR), consisting of an extracellular portion, responsible for antigen recognition and an intracellular portion, responsible for signal transduction.

Studies on infectious complications related to CAR-T cell therapy are scarce and difficult to interpret because of their retrospective single-center design and because of differences in patients’ characteristics, CAR-T cell therapy doses and administration schedules, antimicrobial prophylaxis use and diagnostic approaches. Although epidemiology of infections after CAR-T cell therapy is still poorly documented, the data collected so far show that patients receiving CAR-T therapy are exposed to an increased risk for infection because of several factors, such as immunosuppression due to the hematological cancer and its previous treatment, lymphodepleting chemotherapy administered before cell infusion, chemokine-mediated cytopenia in patients with cytokine release syndrome (CRS) and immune effector-cell–associated neurotoxicity syndrome (ICANS) [28]. Furthermore, CD19 CAR-T cells may deplete normal CD19 B cells in the host, potentially causing prolonged B-cell aplasia and hypogammaglobulinemia [29].

Cytopenia in CAR-T patients is described in several studies [29–32]: In the literature, neutropenia is the most frequent cytopenia (94% of patients), followed by thrombocytopenia and, less frequently, anemia. Recent studies also reported high rates of severe lymphopenia (< 200 cells per mm^3^) in almost 80% of patients [31, 33]. Cytopenia is often associated with an increased risk of infections, in particular protracted and profound neutropenia increases the risk of bacterial and fungal infections, while B-cell aplasia can determine hypogammaglobulinemia, which is associated with infections from encapsulated bacteria [34].

Chronic B-cell aplasia, and resultant hypogammaglobulinemia, is an expected on-target toxicity of successful CD19-directed CAR-T cell therapy. As long as CAR-modified T cells persist, B-cell aplasia continues, which provides what appears to be a highly accurate pharmacodynamic marker of CAR function. Although immunoglobulin replacement mitigates most infectious complications, longer follow-up is needed to assess late toxicity of B-cell aplasia [35].

In the majority of these patients, cytopenia persists several weeks after the infusion: In the literature, 27% of patients with ongoing remission had persistent grade 3 or 4 cytopenia at 1 year and 11% at 2 years [36].

Personal history of infection is also important to stratify the risk of future events: Patients with a precedent invasive mold infection are at high risk for recurrence, as are patients with bacterial or viral infections, in particular *Cytomegalovirus*. Therefore, these patients should be closely monitored and possibly offered antimold and antiviral prophylaxis [37].

It is still uncertain if patients with a history of hepatitis B infection are at high risk of reactivation after CAR-T therapy: There are few data in the literature, mainly series of patients with Non Hodgkin B lymphomas and no substantial reactivation of hepatitis B infection was seen in the setting of antiviral prophylaxis [36, 38, 39]. Therefore, long-term antiviral prophylaxis is prudent.

As far as fungal infections are concerned, the rate of invasive mold infection (IMI) after CAR-T cell therapy appears to be around 1–7% and they are often caused by resistant molds [40]. Haidar et al. [41] reported neutropenia and CRS as risk factors for IMI which occurred with an incidence of 3% in their study. Given the paucity of data, is unknown whether CAR-T recipients may benefit from antimold prophylaxis, thought guidelines recommend prophylaxis when rate of infections is more than 8%.

To correctly stratify the risk of infection, in general, is also important to consider the kind of CAR-T cell therapy used: Fourth generation CAR-T cells, for example, might not require the use of aggressive lymphodepleting chemotherapy, thus decreasing the risk of infection [42]. Lymphodepleting conditioning chemotherapy, indeed, also increases the risk of infection of CAR-T recipients: Cyclophosphamide is known to cause neutropenia, and fludarabine is also a risk factor for opportunistic infections [28].

On the other hand, differences in CAR-T cell products and how those relate to the degree and kinetics of myelosuppression and immunodeficiency recovery, remain undefined. While awaiting further information, it might be prudent to use the same anti-infectious prophylaxis strategies despite the type of CAR-T product [28].

Infectious complications in CAR-T patients might be also related to CRS and ICANS and its treatment. Usually most early infections in CAR-T patients occurs after CRS onset: Cytokine release induces endothelial damage, which could initiate or facilitate infectious processes; moreover, the use of tocilizumab and steroids can predispose patients to infections trough the dysregulation of innate and adaptive immune response [28, 43].

The range of infectious complications in patients with CRS or ICANS goes from mild bacterial and viral infections to life-threatening sepsis often caused by opportunistic microorganisms [28].

In Table 4, we summarized infectious complication in CAR-T cells therapy, relying on the latest studies published so far [29–32].

**Table 4:** Infectious complication in CART-cells therapy

References	Patients	Age	Days post CART infusion	All infections	Bacterial infections	Viral infections	Fungal infections
Hill et al. [30]	133 (47 with ALL)	Adults	< 28 days	23%	17%	8%	3%
			> 28 days	14%	7%	9%	2%
Park et al. [31]	53	Adults	< 30 days	42%	30%	9%	8%
			> 30 days	31%	16%	28%	3%
Vora et al. [29]	83 (81 with ALL)	≤ 26 years old	< 28 days	40%	18%	19%	1%
			> 28 days	17%	6%	11%	0%
Cordeiro et al. [32]	86 (26 with ALL)	Adults	> 90 days	61%	27%	12%	5%
Maude et al. [48]	75	≤ 21 years old	< 90 days	43% (upper respiratory tract 3%)	Septic shock 3% *Staphylococcus* 3%	*Rhinovirus* 3% *RSV 3*% HHV-6 encephalitis 1%	Systemic mycosis 1%

Park et al. [31] examined infections occurring within the first 180 days in 53 adult patients with relapsed or refractory B-ALL treated with CD19-targeted CAR-T cells.

During the first 30 days after the infusion, 26 infections developed in 22 patients (42%); all but 4 infections occurred, while the patient was neutropenic. Bacterial infections (30%) predominated and included 8 bloodstream infections, 1 intraabdominal infection, 4 cases of *Clostridium difficile* diarrhea, 2 pneumonias, 1 pyelonephritis and 1 chest wall abscess. Interestingly, 54% of all bacterial infections diagnosed within the first 30 days after the infusion were due to resistant organisms. These included 3 vancomycin-resistant *Enterococcus* (VRE), 2 extended spectrum beta-lactamase (ESBL) producing *Escherichia coli*, 1 multidrug resistant *Pseudomonas aeruginosa* and 1 carbapenemase producing *Klebsiella pneumonia* infection. Four invasive fungal infections occurred in patients receiving micafungin prophylaxis. These included 1 case of *Saccharomyces cerevisiae* fungemia, 2 of probable invasive pulmonary aspergillosis and 1 of proven pulmonary mucormycosis. There were 2 viral reactivations (1 *Herpes simplex* and 1 *Varicella zoster virus*); the case of shingles occurred in the absence of acyclovir prophylaxis. After the 30th day post infusion, 10 of 31 patients (31%) had 15 infections, which included 9 viral (8 respiratory viruses, 1 *BK* viruria), 5 bacterial and 1 fungal. Three patients died of infectious complications during the study period.

The most important risk factor for infection in this study was CRS: CRS grade 3 or higher was significantly associated with and increased risk of infections, and it was not demonstrated whether tocilizumab or corticosteroids used to treat high-grade CRS increases the risk of infection independent of CRS [31].

In the study of Vora et al. [29], the authors examined the epidemiology and risk factors for infections occurring in the first 90 days after CAR-T infusion in 81 patients with relapsed or refractory B-ALL (and 2 patients with other lymphoproliferative diseases).

In the first 28 days post CAR-T cells infusions, 40% of patients had an infectious event: 54% bacterial, including 16 sepsis (mostly caused by gram-positive bacteria) and 49% had viral infection, the majority of which were due to respiratory viruses [29].

After 28 days post CAR-T cells infusions 17% of patients had an infectious event: The majority of infections were viral (58%), all due to respiratory viruses; only 5 patients had bacterial infections and there were no fungal infections. 45% of the infections were severe, most of which were bacterial, while viral infection were often mild; 16% of infections were life-threatening and only one was fatal, due to septic shock with *Aeromonas hydrophila* 51 days post CAR-T infusion [29].

Risk factors for infections were CRS grade (a higher proportion of patients with higher-severity CRS had infections, 62% of patients had grade 3 CRS or higher, but these data were not statistically significant), neutropenia (90% of patients with bacteremia were neutropenic) and hypogammaglobulinemia [29].

In the study of Hill et al. [30], the authors analyzed the epidemiology of infections during the first 90 days after CD19 CAR-T cell immunotherapy in 133 patients with relapsed or refractory B-cell malignancies, 47 of which with B-ALL, and identify factors that predispose them to a higher risk of infection.

Twenty-three percent of patients had an infection in the first 28 days after the infusion, mostly were bacterial (17%), 4 of which were due to gram negative organism with fluoroquinolone resistance; 8% of infection were viral and only 3% were invasive fungal infections, which occurred in patients with severe CRS requiring tocilizumab and/or corticosteroids [30].

After the first 28 days after the infusion, only 14% of patients had an infection, mostly due to viruses (9%), 89% of which involved the upper respiratory tract, 7% of patients had a bacterial infection and only 2% a fungal infection. Among patients with late infections, persistent disease and neutropenia were found in 48% and 22%, respectively. Patients with the highest risk of infections were those with ALL, who receipt 4 or more prior antitumor treatment regimens and receipt a higher CAR-T cell dose (more than 2 × 10^7^ cells per kg) [30].

Cordeiro and al published an interesting study in 86 patients who undergone CAR-T, 26 of which had ALL [32]. After 90 days from the infusion, 61% of patients had an infection, mostly (48%) of the upper respiratory tract, while 23% of the lower respiratory tract. 60% of infections were bacterial and 31% were viral, mostly respiratory viruses, only 9% were fungal (2 *Aspergillus*, 1 *Candida* and 1 *Coccidioides*) [32].

Although direct comparison between several studies is limited by differences in methods and periods of observation, the data collected so far suggest that infections in CAR-T patients are more frequent during the first month after the infusion and they are mostly bacterial. After the first 30 days, viral infections are predominant and they usually have a better prognosis, while fungal infections, in general, are less frequent and they are often associated with risk factors such as neutropenia [44–47].

In the pediatric population, CAR-T therapy is associated with a high rate of complete remissions (about 90%), but also with a high incidence of side effects, mainly the cytokine release syndrome (88% of patients) and neurological toxicity [46].

Regarding infectious complications, about 36% of pediatric patients with ALL treated with CAR-T experience febrile neutropenia and 45% of these patients have an infectious event, most frequently bacterial. The majority of infections involves the respiratory system; on the other hand, severe grade 3 or 4 infections are usually associated with either neutropenia or prolonged lymphopenia and they are frequently of fungal or viral etiology [48].

Nowadays, SARS-CoV2 plays a crucial role in the epidemiology of infectious diseases, in particular in hematological patients, which are at high risk of severe disease and mortality [49]. Prevalence of SARS-CoV2 infection in CAR-T patients is almost 5%, definitively higher than the 0.1% reported in the general population, with a median onset of 5–6 month after the infusion [50]. Viral infections are indeed more common after day 30 from the infusion, when lymphopenia and hypogammaglobulinemia become critical [51].

Patients receiving CAR-T therapy are more susceptible to COVID infection and prolonged viral clearance time because they often have delayed cytopenia and impaired immune reconstitution and because they frequently have progressive hematologic disease [50].

Most of these patients develop severe infection (67%), with 43% requiring admission to ICU, while 10% develop an asymptomatic form of disease and 20% a mild one [50].

Risk factors associated with more severe cases have not been clearly identified, but having many comorbidities, progressing hematologic disease, and prolonged cytopenia are thought to be variables that may correlate with a more unfavorable course [50].

Mortality is around 30%, higher than general population (from 0.1 to 9.4% across the different countries) [50].

## Conclusions

Outcome in Acute Lymphoblastic Leukemia has shown a steady improvement, however, despite the advances in disease outcome, treatment-related toxicity remains unacceptably high.

Monoclonal antibody-based therapies are well tolerated in ALL patients, and they combine their high efficacy with a lower burden of infectious averse events, in particular for fungal and viral infection, representing an important resource in ALL treatment.
